# Ribosome profiling enhances understanding of mycobacterial translation

**DOI:** 10.3389/fmicb.2022.976550

**Published:** 2022-08-04

**Authors:** Elizabeth B. Sawyer, Teresa Cortes

**Affiliations:** ^1^School of Life Sciences, University of Westminster, London, United Kingdom; ^2^Pathogen Gene Regulation Unit, Instituto de Biomedicina de Valencia (IBV), CSIC, Valencia, Spain

**Keywords:** ribosome profiling, *Mycobacterium tuberculosis*, non-canonical translation, translation initiation, omics

## Abstract

A recent addition to the -omics toolkit, ribosome profiling, enables researchers to gain insight into the process and regulation of translation by mapping fragments of mRNA protected from nuclease digestion by ribosome binding. In this review, we discuss how ribosome profiling applied to mycobacteria has led to discoveries about translational regulation. Using case studies, we show that the traditional view of “canonical” translation mechanisms needs expanding to encompass features of mycobacterial translation that are more widespread than previously recognized. We also discuss the limitations of the method and potential future developments that could yield further insight into the fundamental biology of this important human pathogen.

## Introduction

**FIGURE 1:**
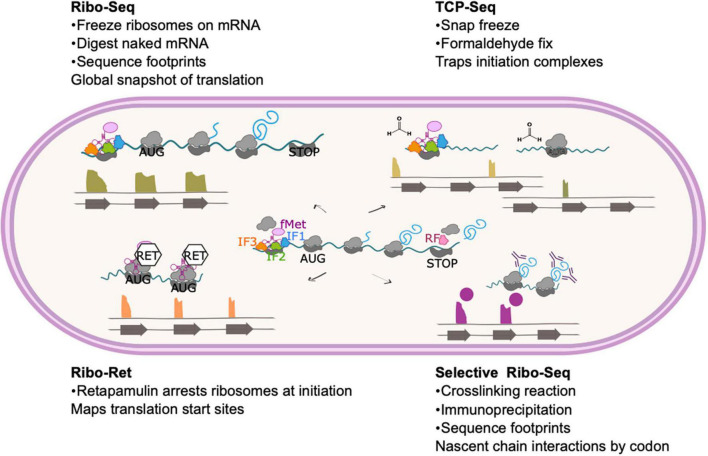
Ribosome profiling method and modifications. The basic ribosome profiling method involves isolation and sequencing of mRNA that is protected from nuclease digestion by ribosome binding. As the central panel shows, these mRNA footprints represent codons across the entire gene often including non-coding regions such as the ribosome binding site. Read length and various *in vitro* and *in silico* selection methods can be applied to enrich for sequences that are more likely to represent active translation rather than non-productive ribosome binding. Variations on this method to select or enrich the library for particular features are shown. TCP-Seq (top right) includes flash freezing and formaldehyde fixation steps to trap initiation complexes. Reads from TCP-Seq therefore often map upstream of the CDS (reflecting formation of the canonical SD initiation complex) or at the start codon (for 70S initiation mechanisms). In ribo-RET (bottom left) cells are treated with retapamulin, which arrests ribosomes at initiation. This method can be used to identify unannotated translational start sites, including those within known coding sequences, such as IRES. Selective ribo-seq (bottom right) involves crosslinking ribosomes to their mRNA and any proteins interacting with the emerging nascent chain, such as trigger factor or other molecular chaperones. Translation complexes that have been trapped during interaction with the target protein are then isolated by immunoprecipitation. The footprints therefore correspond to mRNA that is translated as the nascent chain interacts with the target, indicating the length of nascent chain required for the interaction.

Thanks to an ever-expanding experimental toolkit, the central dogma of molecular biology can now be investigated at every level—from the genome, to the transcriptome, to the translatome, to the proteome—and the regulatory mechanisms operating at all levels of gene expression probed. This review discusses insights uncovered by the ability to study the translatome using ribosome profiling, one of the newest additions to the -omics toolkit. It had long been recognized that there is a poor correlation between the abundance of mRNA transcripts and their corresponding protein concentrations in the cell (Maier et al., 2009; Vogel and Marcotte, 2012; Haider and Pal, 2013; Cortes et al., 2017), but how could this be resolved and the factors underlying it revealed? In 2009 Ingolia and co-workers described an elegant method by which fragments of mRNA that were undergoing active translation could be isolated from a cell and sequenced (Ingolia et al., 2009). The method was developed in eukaryotes and optimization for prokaryotes required a deep understanding of the process of translation and factors affecting it to ensure reproducibility and rule out or mitigate for experimentally induced artifacts (Becker et al., 2013; Subramaniam et al., 2014; Woolstenhulme et al., 2015; Hwang and Buskirk, 2017; Mohammad et al., 2019). In spite of these challenges, many important discoveries about bacterial translation have been made. Some methodological developments that enable investigation of different steps of translation are summarized in Figure 1.

A comprehensive review of the contribution of ribosome profiling to understanding bacterial translation in general is beyond the scope of this review. Rather, we will highlight a few key insights that are of particular interest or relevance.

### Insights about initiation

**FIGURE 2:**
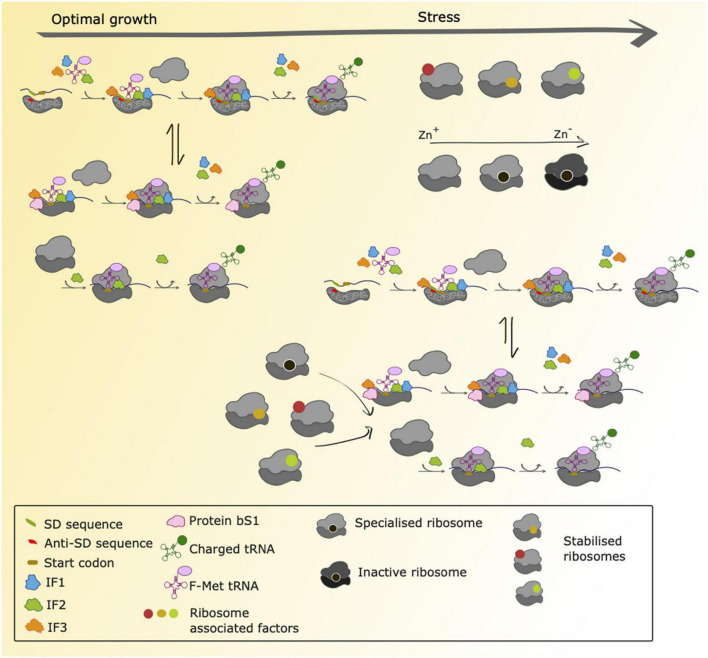
Proposed scenarios for translation initiation in *M. tuberculosis* during normal growth and stress adaptation. During conditions of normal growth, *M. tuberculosis* uses both canonical initiation mechanisms (SD-aSD interaction) as well as alternative initiation mechanisms that are still uncharacterized. These could involve ribosomal protein bS1 to initiate translation of transcripts lacking a strong SD sequence within the 5′ UTR and 70S-mediated initiation of transcripts lacking a 5′UTR as previously characterized in *E. coli*. Ribosome profiling studies have shown that both canonical and non-canonical transcripts are robustly translated in *M. tuberculosis* (Sawyer et al., 2021), suggesting that mechanisms for canonical and non-canonical translation initiation are in place and that there is an equilibrium between both (represented with the arrows in the Figure). During conditions of stress there are ribosome associated factors that could bind with ribosomes to stabilize them. Furthermore, it has been shown that during conditions of zinc deprivation, specialized ribosomes containing paralogs of ribosomal proteins are formed and are translationally active. As conditions of zinc depletion increase, these ribosomes become inactive. We hypothesize that during conditions of stress, *M. tuberculosis* temporarily relies on alternative initiation mechanisms to sustain protein synthesis, and that these mechanisms might be favored by specialized and/or stabilized ribosomes.

Although translation initiation, elongation, termination and ribosome recycling are all amenable to regulation, most control mechanisms described to date operate during the initiation steps. During initiation, the mRNA is recruited to the small ribosomal subunit and the open reading frame (ORF) correctly positioned along with initiation factors, the initiator aminoacyl tRNA*^fMet^*, GTP and eventually the large ribosomal subunit (Figure 2).

Binding of the small ribosomal subunit to the mRNA for translation initiation is traditionally achieved through the base pairing of the Shine-Dalgarno (SD) sequence present in the 5′ untranslated region (UTR) of the mRNA to the complementary anti-Shine-Dalgarno (aSD) region of 16S ribosomal RNA (rRNA) (Shine and Dalgarno, 1974; Figure 1). Nevertheless, alternative mechanisms of initiation operate when mRNA molecules lack an SD sequence. For mRNA molecules that have an SD-less 5′ UTR, interactions between the ribosomal protein bS1 and AU-rich elements in the UTR facilitate formation of the pre-initiation complex (Boni et al., 1991; Komarova et al., 2005; Figure 2). Where mRNA molecules lack a 5′ UTR entirely (referred to as “leaderless”), 70S ribosomes bind at AUG codons equipped with a 5′ phosphate group (Moll et al., 2004; Giliberti et al., 2012; Figure 2). To trap early intermediates of translation, including the pre-initiation complex, and better study every stage of translation, modifications to the ribosome profiling method have been developed. Translation complex profile sequencing (TCP-seq) involves a formaldehyde crosslinking step that fixes transient ribosome/subunit complexes to their mRNAs enabling footprints from initiation complexes to be isolated and sequenced (Archer et al., 2016; Shirokikh et al., 2017; Figure 1). TCP-seq has revealed dynamic rearrangements of the 30S subunit and mRNA during initiation, reflected in different mRNA read lengths (Archer et al., 2016) and the impact of ribosome assembly defects on binding initiation factor 3 (Sharma and Anand, 2019).

The traditional view of the mechanism by which mRNA recruitment and positioning occurs, described by Shine and Dalgarno (1974), has been subjected to much interrogation. However, dissecting out the contribution of SD—aSD interactions from other features of the mRNA and activity of the ribosomal protein bS1 to reach a more refined understanding of the role of each of these features in defining the bacterial proteome has proved challenging (Li et al., 2014; Schrader et al., 2014; Del Campo et al., 2015; Li, 2015). Recently, Saito and co-workers performed ribosome profiling using aSD mutant ribosomes to address the role of the SD-aSD interaction without perturbing mRNA sequences or structures (Saito et al., 2020). Through a series of elegant experiments, their results revealed that although mRNA features other than the SD sequence are able to render complexes initiation-competent, there is a linear correlation between SD strength and translation efficiency (Saito et al., 2020). These findings are in good agreement with previous work that investigated the sequence and structural properties of mRNA in the vicinity of the start codon (O’Donnell and Janssen, 2001; Li et al., 2012; Reeve et al., 2014; Duval et al., 2015; Hockenberry et al., 2017).

### Elongation

At first glance, elongation may appear to be the least interesting and adaptable phase of translation as the ribosome proceeds with aligning tRNAs on the appropriate codons, catalyzing peptide bond formation and translocation to the next codon. However, variations in elongation rate both reflect and impact upon the state of the cell (Dai et al., 2016) and the quality/fidelity of the polypeptides produced (Holm, 1986; Shah and Gilchrist, 2010). The greatest contributor to variable elongation rates is codon bias, the correlation between the abundance of particular tRNAs and the frequency of their cognate codons within the mRNA (Kurland, 1991; Plotkin and Kudla, 2011). The presence of charged amino acids in the nascent polypeptide has also been linked to variation in elongation rates though interactions between the peptide and surface of the ribosome exit tunnel (Riba et al., 2019). Additionally, co-translational folding of the polypeptide chain and translation rate impact each other (O’Brien et al., 2012; Ciryam et al., 2013). These factors are “hard-wired” into the process; whether there are additional factors that allow for dynamic modulation of elongation rate is still under investigation.

In translation, there is a mechanistic trade-off between elongation rate and translational accuracy (Wohlgemuth et al., 2011). To ensure fidelity, it is not necessary to globally slow translation; cells may compromise by slowing translation only at structurally or functionally important residues while sacrificing accuracy for speed at other residues (Doerfel et al., 2013). Ribosome profiling can reveal pause sites where such strategic slowing occurs. The poor resolution of ribosome profiling in bacteria compared to eukaryotes has presented technical challenges, but using known pause motifs (consecutive proline residues, and comparing results from wild-type and strains lacking the elongation factor required for rapid translation of polyproline stretches, EFP) Woolstenhulme et al. were able to adapt the ribosome profiling method to obtain higher resolution data enabling them to characterize pausing in *E. coli* (Woolstenhulme et al., 2015). With such methodological advancements it should be possible to identify additional pausing sites and search for patterns across the genome, relating the findings to what is known about codon conservation and structurally and functionally important sequence elements.

### Termination

Termination, which in bacteria is controlled by three release factors (RFs), is necessary for translational accuracy, especially for genes in operons, and to recycle ribosomes for further rounds of translation. Ribosome profiling studies in *E. coli* have shown read-through beyond the stop codon in more than 50 genes with variable consequences—in some cases read-through expands the protein repertoire and in others produces deleterious proteins (Baggett et al., 2017). The antimicrobial peptide apidaecin, inhibits termination of translation. Ribosome profiling revealed that apidaecin arrests ribosomes at stop codons generating queues of trailing ribosomes. Inhibition at stop codons sequesters RFs, which in turn leads to read-through on other mRNA molecules resulting in proteomic dysregulation. Because the RFs have been sequestered by the apidaecin-stalled ribosomes, neither tmRNA nor *arfA* ribosome rescue mechanisms are able to recover translation (Mangano et al., 2020).

How applicable are these findings to other species of bacteria? It is clear that translation is a more dynamically regulated and highly influenced process than previously understood. Whilst some of the findings described above may be more faithfully reproduced in mycobacteria than others, it seems reasonable to hypothesize that these and/or other mechanisms that expand the “canonical” view of translation are likely to operate. With this in mind, we now turn to key findings from ribosome profiling in mycobacteria.

## Insights from ribosome profiling in mycobacteria

Despite technical challenges (discussed below) ribosome profiling has been performed in mycobacteria, leading to several important discoveries.

### Initiation mechanisms

As discussed above, ribosome profiling has contributed to understanding the role of the SD-aSD interaction in initiation of translation in *E. coli*. Approximately 25% of mRNA molecules expressed by *Mycobacterium tuberculosis* lack a 5′ untranslated region (UTR) altogether and a further 36% have a 5′ UTR that lacks a strong SD sequence (Cortes et al., 2013; Shell et al., 2015). Ribosome profiling revealed that these SD-lacking transcripts are robustly translated under both optimal growth and nutrient starvation conditions (Sawyer et al., 2021; Figure 2). Although nucleotide resolution of ribosome position has not yet been achieved in ribosome profiling in *M. tuberculosis*, metagene analysis of 3′-aligned reads enabled identification of a consensus 14 nucleotide offset to the start codon in the ribosomal P-site. Comparison of the ribosome densities around the start codon between the different mRNA categories (canonical SD, no 5′ UTR and 5′ UTR lacking an SD sequence) revealed striking differences indicating different initiation mechanisms were likely operating on different types of transcripts (Sawyer et al., 2021) consistent with alternative models proposed for initiation (Boni et al., 1991; Moll et al., 2004; Komarova et al., 2005; Figure 2).

Unlike genes lacking a 5′ UTR or a SD sequence, the metagene profiles of SD genes showed a clear increased ribosome density around the start codon, which was proposed to reflect assembly of ribosomes on the mRNA and pausing due to SD-aSD binding and start codon location. To assess whether SD initiation correlated with increased rates of translation across the whole gene, the 5′ UTR was assigned a score based on the number of matches to a consensus SD sequence (obtained from the *M. tuberculosis* 16S rRNA aSD sequence) and the ribosome occupancy (indicative of translation rate) across the whole gene compared across SD-match scores. Genes with high Watson-Crick complementarity to the aSD had statistically significant higher ribosome occupancies than genes with fewer matches (Sawyer et al., 2021). Understood within the context of the additional influences of mRNA structure, degradation rate and other features in the vicinity of the start codon, these results suggest that despite the compensatory and competing mechanisms at play, the canonical SD-aSD initiation model is still highly relevant in influencing the translation rate of several genes, in agreement with the findings of Saito et al. in *E. coli* (Saito et al., 2020).

### Translational fidelity

Translational misreading has been shown to increase in *M. smegmatis* during stationary phase and in the presence of streptomycin (Leng et al., 2015). Whilst translational fidelity is essential for life, errors that increase phenotypic diversity may play a role in adaptation and survival under certain stress conditions (Ribas de Pouplana et al., 2014). Indeed, a recent study exploring the divergence of codon usage in functionally important genes in *M. tuberculosis* found that selective pressure drives codon usage, with implications for understanding evolutionary divergence, the emergence of antibiotic resistance mutations and ultimately treatment outcomes (Li et al., 2022). Evaluating genome-wide translational fidelity obviously relies on gene annotation. However, an additional source of proteomic expansion/diversity is the translation of as-yet unannotated genomic elements that have not been predicted by algorithms as they lack features commonly required for identification as a gene (ORF length, SD sequence, promoter etc.).

A recent ribosome profiling study in *M. tuberculosis* identified many robustly translated short ORFs that had not previously been described (Smith et al., 2022). To achieve this, Smith et al. used a modified form of ribosome profiling, Ribo-RET, in which bacteria are treated with retapamulin to freeze translation during initiation (already elongating ribosomes are unaffected by retapamulin treatment) (Figure 1). Limiting the ribosome footprint signals to start codons in this way enables identification of overlapping ORFs, including those positioned in-frame with known ORFs (i.e., alternative isoforms of annotated genes) (Meydan et al., 2019; Weaver et al., 2019). Smith et al. identified thousands of robustly translated small ORFs, including hundreds that are isoforms of known proteins. Mass spectrometry, initiation reporter systems, western blotting and nucleotide composition analysis were then used to identify which of these novel polypeptides conferred fitness benefits and which may be due to translational noise. Ninety new ORFs, with a median length of 52 amino acids, showed all the hallmarks of being functional (Smith et al., 2022). Further characterization of these small proteins may lead to increased understanding about the role of proteomic diversity in *M tuberculosis* fitness and adaptation. This is in good agreement with ribosome profiling studies in *M. smegmatis* that identified many new genes encoding small proteins likely to play novel roles in diverse cellular processes (Shell et al., 2015).

### Specialization and heterogeneity

Translational accuracy not only involves matching codon-anticodon pairs correctly in the appropriate frame of an mRNA transcript that is robustly (as opposed to “noisily”) translated, but also on the “second genetic code”—the correct aminoacylation of tRNAs [the complexity of this system and its evolution are reviewed in Woese et al. (2000)]. In keeping with the idea that increased phenotypic diversity confers bacterial survival benefits under certain conditions, it has been shown that mistranslation is associated with increased resistance to rifampicin, a first-line antibiotic in the treatment of tuberculosis (Javid et al., 2014; Su et al., 2016).

The *gatCAB* locus in *M. tuberculosis* encodes a heterotrimeric enzyme required for correct decoding of glutamine codons during translation (Curnow et al., 1997). Clinical isolates with mutations in these genes have increased mistranslation and higher rifampicin tolerance compared with wild-type strains (Su et al., 2016). A recent study by Chaudhuri et al. found that the aminoglycoside kasugamycin decreases mistranslation and increases susceptibility to rifampicin in *M. tuberculosis* (Chaudhuri et al., 2018) suggesting that increasing translational fidelity may reduce mycobacterial adaptation and make it easier to treat infection. A ribosome profiling study in *E. coli* found that kasugamycin treatment reshapes the bacterial translatome (Lange et al., 2017) but as the study focused on the influence of the 5′ UTR and gene families associated with kasugamycin tolerance rather than mistranslation rates, further experimentation and analysis is needed to determine the precise mechanisms underlying these observations. As the *E. coli* transcriptome contains very few mRNA molecules that lack a 5′ UTR, perhaps the interplay between kasugamycin, UTRs and mistranslation would be better observed in a mycobacterial model.

Just as proteomic diversity enables rapid environmental adaptation without genomic alteration, heterogeneity in ribosomes themselves is associated with preferential translation of particular subsets of genes (Byrgazov et al., 2013; Chen et al., 2020). Under conditions of zinc starvation, mycobacteria express alternative ribosomal proteins that lack the cysteine-rich motifs of their paralogs (Figure 2; Prisic et al., 2015; Dow and Prisic, 2018). Javid and co-workers used ribosome profiling to reveal that in *M. smegmatis* these alternative ribosomes are not only translationally active but have distinct translational profiles compared to their canonical counterparts (Chen et al., 2020). The alternative ribosome operon contributes to bacterial iron homeostasis *via* a mechanism that is yet to be fully elucidated but is likely to involve regulation of membrane protein and secretion apparatus synthesis. Thus, a picture of translation as a more complex and dynamically regulated process than is traditionally considered, is beginning to emerge.

### Ribosomes and stress response

When bacteria are exposed to environmental stress, their ribosomes may act as either sensors, targets or responders to the change in conditions. A full discussion of the mechanisms underlying these processes is beyond the scope of this review and has already been described by Starosta et al. (2014) and Cheng-Guang and Gualerzi (2021) so here we will focus on how ribosome profiling has contributed to understanding the interplay between ribosomes and cellular stress.

Ribosome hibernation is a widespread bacterial stress response. In *E. coli* this response takes two forms: long hibernation promoting factor (HPF)-mediated ribosome dimerization to form inactive 100S particles (Wada et al., 1995; Maki et al., 2000; Franken et al., 2017); and the stabilization of 70S ribosomes by ribosome associated inhibitor A (RaiA) (Agafonov et al., 1999; Vila-Sanjurjo et al., 2004). Although ribosome dimerization has been described in many species of bacteria (Ueta et al., 2010, 2013; Puri et al., 2014; Kline et al., 2015; Flygaard et al., 2018), it does not appear to occur in mycobacteria, which rather adopt the strategy of 70S stabilization, preventing the dissociation of ribosomes into their 30S and 50S subunits (Trauner et al., 2012). In this context, the upregulation of genes that have been predicted to associate with ribosomes may be associated with stabilization (Figure 2). The genes *Rv1738*, a putative HPF (Bunker et al., 2015); *Rv3241c*, which encodes RafS, a homolog of an *M. smegmatis* ribosome hibernation protein (Trauner et al., 2012; Mishra et al., 2018); and *Rv2632c*, which is related to *Rv1738* (Bunker et al., 2015), were all significantly upregulated at the level of the translatome during conditions of nutrient starvation in *M. tuberculosis* (Sawyer et al., 2021).

Mycobacterial ribosome stabilization has implications both for the global level of translation and for which subsets of genes are most affected by this modification. The translation of leaderless (5′ UTR-lacking) mRNA molecules is initiated by assembled 70S ribosomes. Under stress conditions in mycobacteria, expression of both leaderless genes and ribosome stabilization factors increases (Cortes et al., 2013; Shell et al., 2015; Grabowska et al., 2021; Sawyer et al., 2021). Ribosome profiling has contributed to understanding the interplay between these phenomena and suggests that by adopting distinct mechanisms of translation altogether with stabilization of 70 ribosomes, *M. tuberculosis* may continue to robustly translate non-canonical mRNAs under different growth conditions (Sawyer et al., 2021; Figure 2).

The question of what triggers ribosome stabilization in mycobacteria has proved challenging to address, particularly in terms of its relationship to zinc remodeling (mentioned above). Recent work by Ojha and co-workers showed that different concentrations of zinc induce remodeling or hibernation and that hibernating ribosomes can be reactivated by zinc supplementation (Li et al., 2020). To date no ribosome profiling studies have looked specifically at zinc depletion, although under the conditions of nutrient starvation described in the various studies zinc concentration must have been low. The impacts of zinc depletion-induced ribosome remodeling and hibernation on the footprint lengths of mRNAs recovered in ribosome profiling may well enhance understanding of mycobacterial ribosomal stress responses.

## Limitations of ribosome profiling in mycobacteria

Undoubtedly, ribosome profiling has already revealed many interesting details about translational regulation in mycobacteria; however, methodological developments are required to push the technique toward higher resolution. The single greatest challenge is applying the refinements that have improved the protocol in *E. coli* to a containment level 3 (BSL3) environment, especially in those were local laboratory regulations might limit the use of these techniques. For example, because most antibiotics exhibit some sequence specificity or interfere with particular ribosomal processes, they do not stall translation instantaneously and uniformly, leading to artifacts. To overcome this, many protocols employ flash freezing to stall translation (Oh et al., 2011; Becker et al., 2013; Mohammad et al., 2019). A variety of flash freezing methods have been described, including rapid vacuum harvesting of cells onto a filter which is then plunged into liquid nitrogen and spraying cultures directly into liquid nitrogen, neither of which are easily adaptable to the BSL3 laboratory. Thus, methods such as chloramphenicol inhibition of translation are still generally used in mycobacterial studies, with additional quality control steps to assess and mitigate any observed artifacts [see Sawyer et al. (2021), for example].

For all ribosome profiling studies, the choice of nuclease is important. Bacterial studies use MNase, which exhibits sequence specificity with a preference for cleavage at A and U nucleotides. For GC-rich organisms like mycobacteria, this leads to inefficiency in mRNA digestion and the footprints recovered may reflect this. Where ribosome profiling can be performed on large enough numbers of cells to generate high read numbers per gene fragment of interest, MNase sequence bias averages out sufficiently to confidently determine the A-site codon (Mohammad et al., 2019). Safety requirements limiting the volume and cell densities of mycobacterial cultures that may be grown mean that these averaging thresholds may not be reached for genes expressed at low levels. This needs to be accounted for in any analysis.

The issue of the number of cells that can be harvested for ribosome profiling also relates to a limitation that currently affects all such studies: that ribosome profiling provides a population measurement that cannot account for differences between sub-populations of cells. Thus, low frequency phenotypes such as persister cells cannot yet be specifically studied. For *M. tuberculosis* where persistence mechanisms are vital for its survival within the host and for disease progression, this is a significant limitation.

## Concluding remarks

Despite these limitations, ribosome profiling experiments yield rich data that can greatly enhance understanding of the process and regulation of translation, a fundamental life process. Insights obtained so far challenge and refine the “canonical” view of translation and suggest more dynamic roles for many of the components of the translational machinery. Like many other biological phenomena, the more the process is studied, the more our models and understandings fall short of fully explaining the data. Given the various trade-offs at play between host and pathogen, adaptation and propagation of infection, treatment and side effects, ribosome profiling offers a means to appreciate nuance and complexity that may lead to more targeted and effective treatments for tuberculosis—which are desperately needed.

## Author contributions

EBS and TC conceived, wrote, and reviewed the manuscript. Both authors approved the submitted version.
